# Long-term neurorehabilitation outcomes of pediatric vs. adult onset acquired brain injury

**DOI:** 10.3389/fneur.2022.981991

**Published:** 2022-12-20

**Authors:** Spring Flores Johnson, Pamela S. Klonoff, Ramaswamy Kavitha Perumparaichallai

**Affiliations:** ^1^The Center for Transitional Neuro-Rehabilitation, Barrow Neurological Institute, St. Joseph's Hospital and Medical Center, Phoenix, AZ, United States; ^2^Focus Neuropsychology AZ, Phoenix, AZ, United States

**Keywords:** pediatric acquired brain injury, acquired brain injury, neurological rehabilitation, functional outcomes, intensive neurorehabilitation

## Abstract

**Background:**

Functional outcomes of intensive neurorehabilitation for pediatric onset acquired brain injury (ABI) are understudied. The extent and pervasiveness of impairments are often uncovered years after an ABI and can worsen over time, leading to a cascade of academic, functional, and psychosocial difficulties.

**Objective:**

To examine the long-term outcomes of survivors with pediatric onset vs. adult onset ABI who completed holistic milieu-oriented neurorehabilitation up to 30 years ago.

**Methods:**

One hundred twenty-three survivors of ABI including a pediatric onset group (*n* = 22) and an adult onset group (*n* = 101) with heterogeneous neurological etiologies who attended holistic, milieu-oriented neurorehabilitation. Productivity, driving, and functional outcomes were evaluated using the Mayo-Portland Adaptability Inventory-4 (MPAI-4) and a psychosocial outcome questionnaire. Treatment for the pediatric onset group started much later than onset.

**Results:**

A one-way analysis of covariance revealed no significant differences between the two groups on the MPAI-4. At the follow-up survey, there was no significant difference between age at onset of injury and productivity status. The average follow-up time was ~8 years (*SD* = 6.28) from time of discharge to the time of the survey. Although there was no significant difference between the two groups for driving at the time of admission, the adult onset group was significantly more likely to return to driving after treatment.

**Conclusions:**

This study demonstrates the positive and enduring benefits of holistic, milieu-oriented neurorehabilitation for survivors of pediatric onset ABI regardless of the time between initial injury and engagement in rehabilitative therapies.

## Introduction

Intensive, holistic neurorehabilitation has ameliorated functional impairments from an acquired brain injury (ABI) since Kurt Goldstein first created a holistic treatment program for brain injured soldiers in World War 1 (1–3). It has been proven to be highly effective for adults with ABI, specifically for increasing their independence, reintegration into the community, quality of life, and even their return to driving (4–7). Further, holistic neurorehabilitation, with a therapeutic milieu component (e.g., a structured group treatment environment), demonstrates an effective approach for those requiring neuropsychological rehabilitation after ABI (7–9). This form of intervention has been used and documented primarily in adult onset ABI populations, but has been rarely utilized for pediatric ABI populations, even though traumatic brain injury is among the leading causes of pediatric trauma and disability (10).

Given the rapid and significant development of critical neural network organization and age dependent changes in brain metabolism during childhood, ongoing brain development and outcomes can be significantly impacted and impaired after an injury (11). Thus, childhood ABI can disrupt developmental trajectories, leading to a cascade of emotional, academic, and psychosocial difficulties (8, 12–14) and the extent and pervasiveness of these challenges are not often identified until much later after the injury (15). Younger children are particularly vulnerable, with more enduring impairments if the ABI is sustained prior to school age (16, 17).

Additionally, more severe pediatric brain injuries are related to poorer outcomes, quality of life, and arrested development 12 and 30 months after injury (16). Cognitively, ongoing impairments in attention, learning, memory, and processing speed commonly impact a child's ability to perform adequately academically (18, 19). Pediatric onset brain injury can also disrupt the development of neural networks associated with social functions, resulting in significant psychosocial challenges (15, 20). These psychosocial difficulties are often long-lasting, persisting into adulthood (21, 22). Behavioral difficulties manifest as disinhibition, aggressiveness, low frustration tolerance, lack of empathy, apathy, emotional lability, depression, anxiety, and limited awareness (14).

Unfortunately, the pediatric population largely remains underserved and understudied within the context of intensive neurorehabilitation (23). One of the biggest hurdles is that the school environment is often designated as the main system of rehabilitative care post inpatient hospitalization and rehabilitation. Essentially, to keep children on an academic course and trajectory, there is little time allotted for students to engage in intensive neurorehabilitation services outside of the academic setting. Given pediatric onset ABI populations' lack of access to intensive, holistic, post-acute neurorehabilitation, it is crucial to understand the potential benefits of this population engaging in these programs.

There is also a common myth that recovery from brain injury plateaus at 2 years after the initial injury (24). However, it has been well-documented that long-term functional gains can be acquired if the patient receives intensive neurorehabilitation, even multiple years after the onset of the ABI, especially with an emphasis on compensation training (7, 25–27).

### Objectives

The specific aim of the present study was to explore the long-term outcomes (e.g., productivity status, driving status, and functional outcomes) of survivors of pediatric onset ABI in comparison to adult onset ABI individuals who engaged in holistic, milieu-oriented neurorehabilitation up to 30 years ago. The average follow-up time was ~8 years (*SD* = 6.28) from time of discharge to the time of the survey.

## Materials and methods

This study protocol was approved by the Institutional Review Board (IRB) at the facility where the study was conducted. The procedures of the study were in accordance with the standards of the IRB.

### Participants

The sample included 123 (22 pediatric onset and 101 adult onset) survivors of ABI with heterogeneous neurological etiologies, who attended holistic, milieu-oriented neurorehabilitation between 1986 and 2016 at the Center for Transitional Neuro-Rehabilitation (CTN), Barrow Neurological Institute in Phoenix, Arizona. They completed one or more neurorehabilitation programs to facilitate: (a) home and community independence, (b) social relationships and quality of life, (c) work re-entry, and/or (d) school re-entry.

Demographic and injury-related information about the participants are presented in Table 1. ABI etiologies included cerebrovascular accident, traumatic brain injury, anoxic injury, and brain tumor. Participants were grouped as pediatric (< 18 years-old) and adult (>18 years-old) based on their age of onset of the ABI. Participants received treatment in the areas of neuropsychology, speech-language pathology, occupational therapy, physical therapy, and recreational therapy. Participants also received services in the areas of psychiatry and nutrition as appropriate. Tenets of holistic milieu therapy are improving patients' (and their caregivers') awareness, acceptance, and realism about the aftereffects of ABI as well as developing and implementing compensations across settings. Interventions are embedded in peer interactions in interdisciplinary groups addressing cognitive, language, physical, emotional, interpersonal, and functional strengths and challenges while engendering bonding and a collaborative working alliance (28).

**Table 1:** Demographics based on onset of injury type (*n* = 123).

**ABI participant demographics**	**Pediatric** **(*n* = 22)**	**Adult** **(*n* = 101)**	***p*-value**
Age at the time of injury, years (mean ± SD)	14.35 ± 5.44	37.38 ± 14.27	< 0.001
Age at admission to neurorehabilitation, years	23.33 ± 8.68	38.80 ± 14.42	< 0.001
Age at the time of survey, years	33.77 ± 12.81	48.65 ± 15.22	< 0.001
Injury to admission duration, months	108.22 ± 142.15	19.03 ± 27.96	0.01
Length of treatment, months	14.85 ± 7.54	12.93 ± 13.56	0.60
Education at admission to neurorehabilitation, years	11.93 ± 1.24	15.06 ± 2.38	< 0.001
Injury type*			0.13
TBI	12 (55%)	62 (61%)	
CVA/AVM	6 (27%)	26 (26%)	
Brain tumor	N/A	8 (8%)	
Other	4 (18%)	5 (5%)	
Sex			0.44
Male	12 (55%)	64 (63%)	
Female	10 (45%)	37 (37%)	
Race/Ethnicity**			0.11
Caucasian	13 (59%)	81 (80%)	
Hispanic	5 (23%)	9 (9%)	
Black/African American	N/A	2 (2%)	
Asian	2 (9%)	3 (3%)	
Other	2 (9%)	6 (6%)	

*There were no CVA/AVM participants in the pediatric onset group and therefore this group was not included in the chi-square test of independence; ^**^There were no Black/African American participants in the pediatric group and therefore this group was not included in the chi-square test of independence.

### Materials

#### The Mayo-Portland Adaptability Inventory-4

The MPAI-4 (29) was completed by participants to measure overall functioning. The MPAI-4 is a 30-item questionnaire designed to investigate physical, cognitive, and psychosocial limitations that commonly occur after ABI. The items are rated using a five-point scale ranging from “no problem” to a “severe problem.” It produces three subscale scores: Abilities, Adjustment, and Participation, as well as a Total Score (29). The MPAI-4 has strong overall person (*r* = 0.88) and item (*r* = 0.99) reliability as well as internal consistency (Pearson Reliability = 0.88). Lower scores indicate higher functionality (29).

#### The long-term outcome questionnaire

The LOQ was developed to obtain specific information about the survivors' living situation, driving status, productivity status (competitive employment, school, homemaker, and/or volunteer work), financial management, social life, leisure, and quality of life (7).

#### Outcome measures

##### Productivity

Productivity status was assessed at the time of program admission, program discharge, and follow-up study participation. Productivity was defined as engaging in part-time or full-time competitive employment, school, homemaking, or volunteering. Unemployment was considered unproductive. Study participants who were retired at the time of the study were removed from the productivity analysis.

##### Driving

Driving status was measured as a dichotomous variable, indicating whether or not the participants were driving at the time of program admission, program discharge, and follow-up study participation.

##### Functional outcome

The MPAI-4 Total score and subscales of Abilities, Adjustment, and Participation were used to determine overall perception of functional status up to 30 years after discharge from neurorehabilitation. Level of functioning was determined by the MPAI-4 Total Score and described as the following: Good Outcome (< 30); Mild Limitations (30–40); Mild to Moderate Limitations (41–50); Moderate to Severe Difficulties (51–60); and Severe Limitations (>60).

### Procedure

The current study employed a survey that was distributed at a 30-year CTN reunion event in October 2016 for survivors of ABI and their caregivers. For those who could not attend the event, phone call and email follow-up continued until December 2018. Participants were given the option to complete the survey in the clinic, over the phone with a member of the research staff, or online. Please see the article by Perumparaichallai, Lewin, and Klonoff for more details about the population and attrition rate of participants (7).

### Data analysis

Between groups comparisons were conducted using two-tailed *t*-tests, chi square (χ^2^) analyses and ANCOVAs. For background and demographic comparisons, *p*-values were consistently interpreted as *p* < 0.05 indicating statistical significance (please see Table 1). A Bonferroni correction was utilized and *p* < 0.01 was considered statistically significant. Covariate analysis was done when appropriate. The SPSS software package, version 27, was used for all statistical analyses.

## Results

### Demographic variables

The pediatric onset and adult onset groups did not significantly differ (*p* > 0.05) in regards to length of treatment, injury type, sex, or race/ethnicity. The adult group had significantly higher levels of education at the time of admission to neurorehabilitation (*p* < 0.001). Additionally, the pediatric onset group experienced significantly longer periods of time between the initial onset of their ABI and admission to neurorehabilitation (*p* = 0.01; please see Table 1).

### Productivity

At the time of the survey, 81% (17 out of 21) pediatric onset survivors of ABI and 90% (77 out of 86) adult onset survivors of ABI were productive (work full-time/part-time, volunteer, homemaker). A chi-square test of independence showed that there was no significant association between age of onset and productivity status, *X*^2^ (2, *N* = 107) = 1.28, *p* = 0.53. This was after removing 15 retired participants from the adult onset group and one retired participant from the pediatric onset group that presented for treatment at the age of retirement (please see Table 2).

**Table 2:** Productivity status at follow-up.

**Productivity status**	**Pediatric** ***n* (%)**	**Adult** ***n* (%)**
Full-time/part-time/school	14 (67)	66 (77)
Volunteer/child-care/homemaker	3 (14)	11 (13)
Unemployed^*^	4 (19)	9 (10)

*1 pediatric onset participant and 15 adult onset participants were excluded from percentage analyses.

### Driving

At the time of admission, 2 (9%) out of 22 survivors of pediatric onset ABI compared to 22 (22%) of 101 survivors of adult onset ABI were driving. A chi-square test of independence showed that there was no significant association between age of onset and driving status, *X*^2^ (2, *N* = 123) = 1.85, *p* = 0.17. However, at discharge from intensive neurorehabilitation, there was a significant difference between survivors of pediatric onset and adult onset ABI (*X*^2^ = 6.88; *p* = 0.01). Seven (32%) of 22 survivors of pediatric onset ABI and 63 (62%) of 101 adult onset survivors returned to driving successfully at the time of discharge. Further, at the time of the survey, 11 (50%) of 22 pediatric onset survivors, and 75 (74%) of 101 adult onset survivors were driving (please see Table 3). This also revealed a significant difference between the two groups (*X*^2^ = 5.05; *p* = 0.02) with the adult group performing better; however, an impressive increase in return to driving or starting to drive was demonstrated for both groups.

**Table 3:** Driving status.

**Driving status**	**Pediatric *n* (%)**	**Adult *n* (%)**
Driving at admission	2 (9)	22 (22)
Driving at discharge*	7 (32)	63 (62)
Driving at survey**	11 (50)	75 (74)

*X^2^ = 6.88; p = 0.01; **X^2^ = 5.05; p = 0.02.

### Functional outcome

A one-way ANCOVA revealed no significant differences between the pediatric and adult onset groups for the productive status on the MPAI-4 Total Score and subscales of Adjustment, Ability, or Participation while controlling for age at the time of the survey and education level at the time of admission (please see Table 4).

**Table 4:** Relationship between acquired brain injury onset and MPAI-4 scales.

**MPAI-4 scales** **at follow-up**	**Pediatric (*n* = 22)** **(mean ±SD)**	**Adult (*n* = 100)** **(mean ±SD)**
Abilities	40.09 ± 15.32	38.65 ± 14.14
Adjustment	36.32 ± 15.18	35.34 ± 12.37
Participation	30.18 ± 17.79	25.39 ± 16.29
Total	38.95 ± 13.57	36.17 ± 14.34

## Discussion

Holistic, intensive neurorehabilitation has been found to be an effective and enduring way to help survivors of ABI return to functioning and independence by focusing on creating an individualized and holistic recovery pathway of bolstering preserved strengths, compensating for ongoing challenges, and generalizing learned strategies to improve independence and community functioning (4–7).

Holistic, intensive neurorehabilitation with a milieu component adds the extra feature of providing a community of collective healing aiding in the development of psychosocial and work skills in tandem with overall adjustment after ABI (28). It is clear that this form of intensive neurorehabilitation has proved extremely beneficial for adults with ABI (7–9).

Even though traumatic brain injury is one of the leading causes of pediatric trauma and disability (10), this population remains vastly understudied and underserved in its participation in intensive neurorehabilitation (23). Additionally, age dependent changes in brain metabolism as well as development of critical organization of neural networks as children grow may influence the response and tolerance to injury impacting long-term neuropsychological functioning (11, 23). Further, younger children are particularly vulnerable, with deficits most apparent when brain injuries are sustained prior to school age (16, 17), causing a host of functional, psychosocial, and academic challenges (8, 12–14).

There are also barriers to engagement in intensive rehabilitation services given academic demands, availability, and time constraints for pediatric survivors of ABI. For school-aged children that have survived a severe ABI, 21% were placed in general/regular education classes and promoted each year despite having significant academic difficulties (30). However, the majority of children receive increased school support services including special education services as the Individuals with Disabilities Education Act and Section 504 of the Rehabilitation act of 1973 mandate (31).

Despite the provision of educational services and support, many childhood survivors of ABI experience serious functional, psychological, and vocational limitations and challenges even through adulthood (8, 31–33). Additionally, although schools are federally mandated to provide these supportive academic services, it does not mean that schools can or should be the main source of rehabilitative care for this population. Unfortunately, this often becomes the case at the expense of long-term functional outcomes (31). Given the necessity for school-aged children to continue expected academic trajectories, this can create a time and accessibility barrier for engagement in intensive, holistic neurorehabilitation programs. It is also important to explore functional outcomes for survivors of pediatric ABI who are unable to engage in intensive neurorehabilitation until much later, even years, after the initial onset of ABI.

The present study aimed to examine the potential benefits of holistic milieu neurorehabilitation for a pediatric population by exploring the long-term productivity status, driving status, and functional status of survivors of pediatric onset ABI in comparison to survivors of adult onset ABI. This study demonstrated the positive and functional impact of participation in holistic, milieu-oriented neurorehabilitation for pediatric ABI survivors even up to 30 years after therapy completion. Specifically, there was no significant difference between the pediatric and adult onset ABI groups in their return to work. Nor was there a significant difference between the two groups in their overall perceptions of functional status with the two groups endorsing a mean level of functioning at mild limitations to good outcomes. This illustrates the benefit of holistic, intensive neurorehabilitation on return to work and functionality for not only adult onset ABI but also for pediatric onset ABI regardless of the time of initial onset of injury to engagement in services. Thus, ideally schools, parents, and holistic, intensive neurorehabilitation programs work together to provide conjoint care focused on a holistic healing, and academic journey to maximize health and functional outcomes.

Although there was a significant difference between the two groups in regards to the ability to return to driving and survivors of adult onset ABI were more likely to return to driving than the pediatric onset ABI group, it is worth noting that 13 out of the 22 survivors of pediatric onset ABI incurred their injury before the age of 17 and only four of these 13 successfully returned to driving. Given this, the procedural learning that is inherent in learning to drive was likely never crystalized making a return to driving or starting to drive after a significant ABI even more difficult for these 13 participants. However, it is still impressive that 50% (11 out of 22) of the pediatric onset group did successfully return to driving or started to drive in the long-term.

### Study limitations

The results of the current study should be interpreted in light of several limitations. First the sample size for those with pediatric onset ABI was low, although consistent with prior research given the limited participation this group typically has with intensive neurorehabilitation given the barriers discussed above (30, 31). Additionally, there was a relative lack of diversity within the study sample. The participants of this study were primarily Caucasian leading to an overall homogeneity of the sample that could affect the overall generalizability of the study. It would be beneficial for future studies to delve further into the how engagement in holistic neurorehabilitation aids with academic functioning and trajectory. Further research is also needed to identify factors that help survivors of pediatric brain injury start to drive or return to driving, specifically age at insult, injury etiology, sociodemographic, cultural, and diversity factors, and the relative contributions of physical vs. cognitive sequelae. Overall, this populations' engagement and functional benefit from participation in holistic neurorehabilitation requires further study in general to create further avenues of functional success for survivors of child onset ABI.

## Conclusions

This study demonstrates the positive and enduring benefits of holistic neurorehabilitation programs for survivors of pediatric onset ABI regardless of the time between initial injury and engagement in rehabilitative therapies. Those who engage in holistic neurorehabilitation, whether the injury was sustained as a child or an adult, demonstrated significant and beneficial gains in their productivity, functional status, and return to driving or started to drive even up to 30 years after discharge. This study implies that it is imperative that clinicians, pediatricians, neurorehabilitation specialists, schools, and parents advocate for pediatric onset ABI survivors to participate in intensive neurorehabilitation programs to enhance their quality of life, including their productivity and functional independence in the community.

## Data availability statement

The datasets presented in this article are not readily available because of ethical and privacy restrictions. Requests to access the datasets should be directed to the corresponding author.

## Ethics statement

This study protocol was approved by the Institutional Review Board (IRB) at the facility where the study was conducted—St. Joseph's Hospital and Medical Center. The procedures of the study were in accordance with the standards of the IRB. Written informed consent to participate in this study was provided by the participants or their legal guardian.

## Author contributions

All authors listed have made a substantial, direct, and intellectual contribution to the work and approved it for publication.
